# Effects of Synthesis Conditions on the Structure and Conductivity of Hydrogen-Substituted Graphdiyne

**DOI:** 10.1021/acsmaterialsau.5c00228

**Published:** 2026-02-04

**Authors:** Karam Eeso, Akriti Sarswat, Zhitao Chen, Cheng-Tien Hsieh, Timothy N. Lambert, Nian Liu

**Affiliations:** † School of Chemical and Biomolecular Engineering, 1372 Georgia Institute of Technology, Atlanta, Georgia 30332, United States; ‡ 1105 Department of Photovoltaics and Materials Technology, Sandia National Laboratories, Albuquerque, New Mexico 87185, United States; § Center for Integrated Nanotechnologies, Albuquerque, New Mexico 87185, United States

**Keywords:** graphdiyne, hydrogen-substituted graphdiyne, synthesis−structure relationship, alkyne retention, thermal transformation, conductivity

## Abstract

This study investigates how synthesis conditions influence the structure and conductivity of hydrogen-substituted graphdiyne (HsGDY). By varying the reaction temperature and solvent, we find that small changes in conditions markedly affect triple-bond retention and electronic continuity. Solid-state ^13^C NMR and Raman spectroscopy reveal that elevated temperatures drive alkyne loss and partial graphitization, with *N*,*N*-dimethylformamide (DMF) promoting faster degradation than pyridine. The resulting decline in alkyne content directly correlates with reduced conductivity, indicating that preserving conjugation is essential for charge transport. These findings clarify how the synthetic environment governs the structural and electronic evolution of graphdiyne frameworks, providing insight into the controlled preparation of conjugated carbon networks.

## Graphical abstract

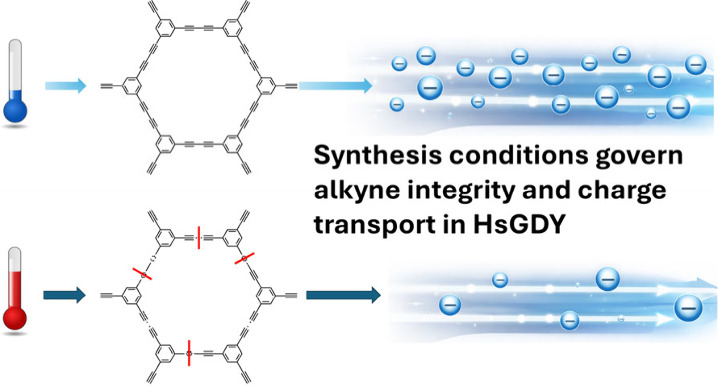

Carbon-based materials have played a central role in the development of advanced functional materials, with particular attention paid to allotropes like graphene, carbon nanotubes, and most recently graphdiyne (GDY). GDY is a two-dimensional carbon network featuring both sp- and sp^2^-hybridized carbon atoms arranged in a π-conjugated framework.^1^ The incorporation of acetylenic linkages introduces unique electronic properties, increased chemical reactivity, and tunable porosity,^2^ making GDY a versatile platform for applications in catalysis,^3–5^ energy storage,^6–14^ energy conversion materials,^15–18^ electronics, and life sciences.^19^

Despite these advantages, the synthesis of GDY and its analogues remains challenging. Subtle changes in the reaction temperature, solvent polarity, or catalytic conditions can dramatically affect the structure and quality of the resulting material. The reaction conditions can influence the extent of conjugation, the retention of triple bonds, and the overall degree of polymerization—all of which are critical to the functional performance of GDY-based systems. However, systematic investigations into how these synthetic variables impact material properties are limited.

As shown in Figure 1, a survey of the literature reveals that reported syntheses occur between room temperature and 120 °C, with significant variation in whether triple bonds are preserved or not. Raman spectroscopy is frequently used to infer the presence of sp-carbon bonding; yet, the signals vary in intensity, and NMR data are often missing altogether. This inconsistency raises questions about the reliability of reported GDY syntheses, as well as the structural determination of the materials being produced.

**Figure 1:**
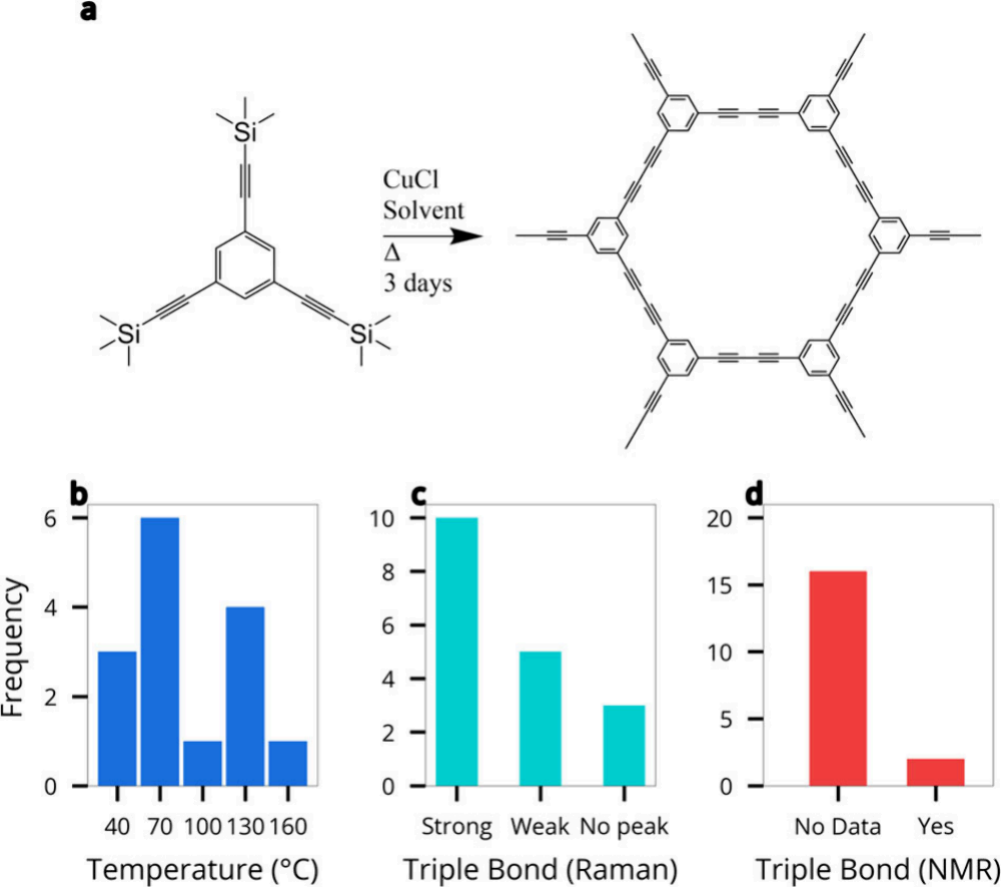
Summary of reported synthesis conditions and spectroscopic analysis methods used in graphdiyne literature.^1,7,8,11–13,20–31^ (a) Synthesis scheme for HsGDY. (b) Histogram showing the distribution of synthesis temperatures across previous studies. (c) Classification of Raman peak quality used to assign the presence of alkyne bonds, categorized as “strong”, “weak”, or “no peak”. (d) Identification of whether ^13^C NMR data were included to confirm triple bond retention. Data compiled and analyzed from refs 8, 12, 13 , and^20–34^ .

In this work, we use hydrogen-substituted GDY (HsGDY) as a model system to probe the influence of synthetic conditions on the structural and electronic properties of graphdiyne frameworks. By varying the reaction temperature and solvent, we explore how these parameters affect triple bond retention, material morphology, and conductivity, a property important to many of GDY’s potential applications. Our goal of this work is to understand how changes in reaction conditions influence the structural, chemical, and electronic properties of GDY-like materials.

We began by preparing HsGDY according to a modified Glaser-type reaction (see Supporting Information).^32^ Briefly, trimethylsilyl-1,3,5-triethynylbenzene (TMS-TEB) (76 mg, 0.2 mmol) and copper chloride (6 mg, 0.06 mmol) were combined in pyridine or *N*,*N*-dimethylformamide (DMF) (2 mL) in a glass vial. The vial was placed in a 40 °C, 75 °C, or 110 °C water bath for 72 h, and then the solid was isolated, washed, and dried. The percent yield and carbon contents are shown in Table S1. Generally, the yield did not change within a given solvent, but pyridine did have an increased yield compared to DMF, which is most likely due to the decrease in solubility of CuCl. Furthermore, XPS at different etching depths shows that low-temperature samples had more silicon, likely due to the incomplete cleavage of the TMS group. In contrast, samples synthesized at higher temperatures show reduced silicon content and increased nitrogen incorporation, likely due to solvent-derived nitrogen incorporation. We first evaluated the transformation of the alkyne structure across different synthesis conditions using a combination of Raman spectroscopy and ^13^C MAS NMR (Figure 2). As shown in Figure 2a, the graphdiyne structure contains four distinct carbon environments: the aromatic C–H and C–C sites (assignments 1 and 2) and the two sp-hybridized alkyne sites (assignments 3 and 4).^11,32,33^ These assignments provide the framework for interpreting chemical changes resulting from variations in the temperature and solvent. Furthermore, each sample will be labeled with a letter to indicate the solvent (“P” for pyridine and “D” for DMF) and a number for the reaction temperature.

**Figure 2:**
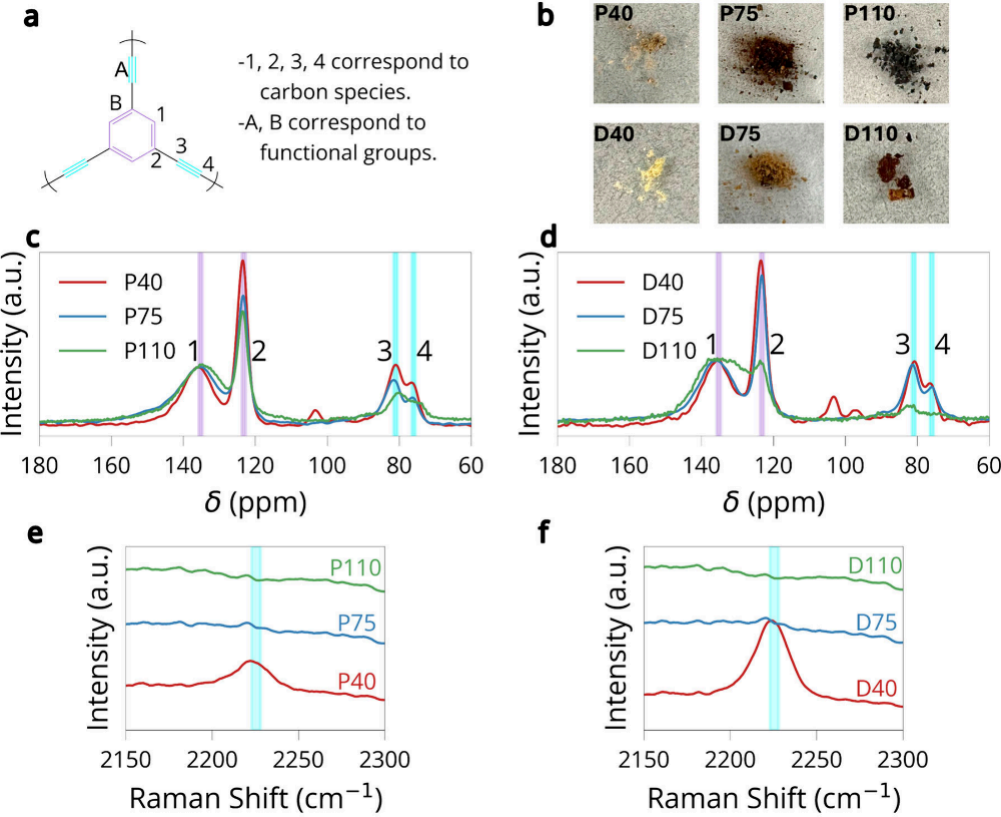
Chemical characterization of graphdiyne frameworks synthesized at different temperatures and in different solvents. (a) Structural assignments of the four main carbon environments used in spectral analysis. (b) Optical images of samples. (c) Solid-state ^13^C NMR spectra for samples synthesized in (c) pyridine or (d) in DMF at 40 °C, 75 °C, and 110 °C. (e) Raman spectra of pyridine-based samples. (f) Raman spectra of DMF-based samples.

The first indication that the synthesis conditions changed the properties of HsGDY is the color of each powder, shown in Figure 2b. The color of the powder darkens as temperature increases in both solvents, but less significantly in DMF. The progressive darkening of the material with increasing synthesis temperature is the first suggestion that partial carbonization and framework disorder arise from alkyne degradation, whereas lower synthesis temperatures preserve the extended π-conjugation associated with intact graphdiyne linkages. In pyridine, the ^13^C NMR spectrum of the sample synthesized at 40 °C displays well-resolved peaks for both aromatic and alkyne carbons. As the reaction temperature is increased to 75 °C and then 110 °C, the intensity of the alkyne peaks at ∼75.5 and 81.8 ppm decreases substantially. A similar trend was observed in DMF, although the extent of degradation is more severe at higher temperatures. For the sample synthesized in DMF at 110 °C, the alkyne signals are nearly undetectable, indicating a dramatic reduction in sp-carbon content. To semiquantitatively assess this trend, we integrated the NMR peak areas corresponding to the alkyne carbons (peaks 3 + 4) and normalized them to the aromatic carbon (peak 2), as shown in Figures S1–S6 and Table S2. The resulting ratios show a consistent decline with increasing temperature in both solvents.

In pyridine, the ratio drops from 0.37 at 40 °C to 0.27 at 110 °C. In DMF, it falls from 0.31 to 0.25 over the same temperature range. This analysis is consistent with the idea that elevated temperatures drive significant consumption of triple bonds and that the extent of this degradation is influenced by the reaction medium.

The effect of the synthesis conditions is also evident in the aromatic region of the ^13^C NMR spectra. In pyridine, increasing the synthesis temperature leads to broadening and downfield shifting of the aromatic peaks at ∼123 and 135 ppm, consistent with chemical restructuring, such as cross-linking or partial graphitization.^34,35^ These changes indicate that the conjugated framework underwent irreversible modifications. In contrast, the samples synthesized in DMF exhibit a more gradual transition. The alkyne peaks remain sharper at 75 °C compared with their pyridine counterparts, and the aromatic signals are less distorted, suggesting that DMF may slow down or suppress some of the side reactions responsible for alkyne loss and structural rearrangement. This may be attributed to differences in solvent coordination, polarity, and interaction with the copper catalyst. Pyridine, being a stronger Lewis base, may promote faster coupling or enable competing pathways that destabilize the sp-carbon framework.

Raman spectroscopy (Figure 2e and 2f) was used to monitor the alkyne region, specifically the C≡C stretching vibration around 2230 cm^–1^.^25,32,33,36^ In pyridine, the sample synthesized at 40 °C displays a discernible alkyne peak that fades at 75 °C and is nearly absent at 110 °C. A similar pattern is observed for DMF: the 40 °C sample shows a large signal, the 75 °C sample retains a weak signal, but the 110 °C sample shows almost no evidence of triple bonds. However, even in samples in which NMR clearly detected triple bond peaks, the Raman spectra are surprisingly weak or featureless in the alkyne region. This discrepancy likely stems from the low Raman scattering cross-section of the alkyne moiety in these extended carbon networks along with broadening effects due to disorder, sample heterogeneity, and potential overlap with background fluorescence. As a result, Raman underrepresented the alkyne content, especially in partially conjugated or amorphous domains. XPS is another commonly used technique to characterize HsGDY in prior studies. The decrease in alkyne bonding with increasing synthesis temperature is further corroborated by quantitative deconvolution of the high-resolution C 1s XPS spectra, which shows a systematic reduction in the sp:sp^2^ carbon ratio (shown in Figures S7 and S8 and Table S1). FT-IR spectra shown in Figure S9 do not show a distinct alkyne stretching mode, which is attributed to the weak infrared activity of C≡C bonds embedded in an extended conjugated carbon framework and to baseline noise inherent to these measurements.

To evaluate how synthetic conditions affected the porous structure of the materials, we analyzed nitrogen physisorption isotherms and pore size distributions (Figure 3). Figure 3a and 3b displays the N_2_ adsorption–desorption isotherms collected at 77 K for materials synthesized in pyridine and DMF, respectively. In pyridine (Figure 3a), all samples exhibit type IV isotherms, characteristic of mesoporous materials, with noticeable hysteresis loops at high relative pressures. The sample synthesized at 40 °C shows the highest uptake, particularly above *P*/*P*_0_ = 0.8, while the sample prepared at 110 °C adsorbs significantly less nitrogen across the full pressure range, suggesting a substantial loss of porosity at higher temperatures. The same trend holds in DMF (Figure 3b), although with key differences. The 75 °C DMF sample shows a dramatic increase in total uptake relative to both 40 °C and 110 °C, with a large hysteresis loop indicative of abundant mesopore formation. In contrast, the 110 °C sample exhibits significantly reduced adsorption, consistent with collapse or densification of the porous network under elevated thermal conditions. These results suggest that while both solvents produced porous materials the evolution of porosity is not directly proportional with temperature and depends critically on the interplay between reaction rate, coupling efficiency, and possible structural rearrangement.

**Figure 3:**
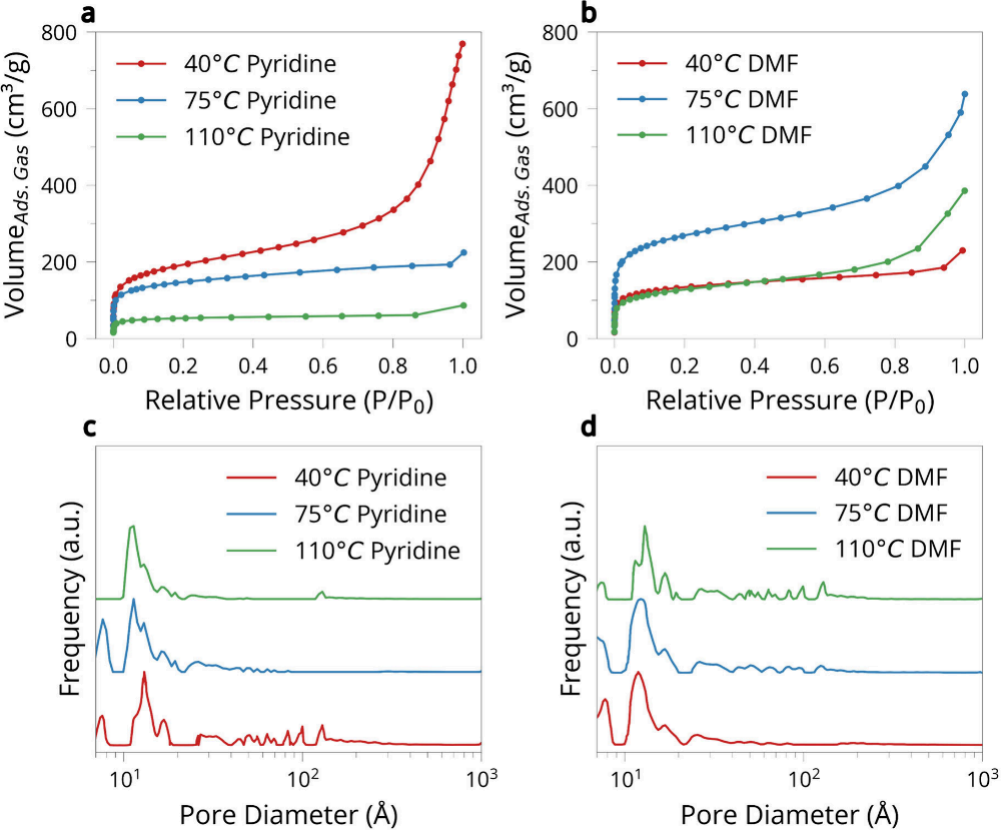
Porosity and structural ordering of graphdiyne materials synthesized in pyridine and DMF. (a) Nitrogen adsorption–desorption isotherms for pyridine samples. (b) Nitrogen adsorption–desorption isotherms for DMF samples. (c) BJH pore size distributions for pyridine samples. (d) BJH pore size distributions for DMF samples as derived from adsorption data.

Quantitative Brunauer–Emmett–Teller (BET) surface area measurements are summarized in Table S2. In pyridine, surface area decreases steadily with increasing temperature, from 676 m^2^/g at 40 °C to just 197 m^2^/g at 110 °C. This trend suggests that higher temperatures drive framework collapse or excessive cross-linking, which may be linked to the same processes responsible for triple bond consumption observed by NMR. In DMF, the surface area reaches a maximum of 958 m^2^/g at 75 °C—significantly higher than the low- and high-temperature samples (482 and 451 m^2^/g, respectively). The steep drop at 110 °C again points to structural degradation under thermal stress.

Figure 3c and 3d shows the BJH pore size distributions derived from the adsorption branches of the nitrogen isotherms. In pyridine (Figure 3c), the 40 °C sample exhibits a dominant pore population in the 10–20 Å range but also displays a broader distribution extending up to ∼100 Å, indicating the presence of larger pores across a wide size range. As the synthesis temperature increases to 75 °C and 110 °C, this broader distribution narrows and shifts toward smaller pore sizes, consistent with progressive pore collapse or thermally induced densification. In contrast, the DMF samples (Figure 3d) exhibit the opposite trend. While the 40 °C and 75 °C samples are mostly confined to smaller pores, the 110 °C sample shows a broader distribution that extends to larger pore sizes. This suggests that a higher synthesis temperature in DMF promotes the formation or retention of larger pores, in contrast to the collapse observed in pyridine.

To assess the degree of long-range structural ordering, we performed powder X-ray diffraction (XRD) on all samples (Figure S10). In pyridine, increasing the synthesis temperature causes a progressive rightward shift of the main diffraction feature from 2θ of ∼19° to ∼22°, suggesting a decrease in interlayer spacing. At 110 °C, a new peak emerged around 11°, indicating the development of an additional periodic motif or structural rearrangement rather than a simple loss of order. A similar trend was observed in DMF, where the primary peak also shifts with temperature, and the low-angle feature at ∼11° becomes prominent at 110 °C. Compared to pyridine, the diffraction patterns in DMF retain slightly more intensity, particularly at 75 °C, suggesting modestly greater structural persistence. Thus, heating promoted rearrangement of the amorphous matrix and not just additional disorder. SEM imaging (Figure S11) reveals significant temperature- and solvent-dependent morphological evolution, with pronounced framework collapse observed for pyridine-derived samples at elevated temperatures, consistent with the corresponding decrease in surface area measured by BET analysis. However, a difference cannot be seen with TEM (Figure S12).

To evaluate the bulk electronic properties of the graphdiyne frameworks, we measured steady-state current flow through the material while it was compressed under 100 MPa using a hydraulic pellet press. Powdered samples were loaded into a steel die, and a fixed voltage was applied across the press anvils, with current recorded over time (Figure S13 and Figure 4c). This setup allows the material to be tested in situ under pressure, ensuring dense packing and intimate particle contact, which minimizes interparticle resistance and highlights the intrinsic electronic response of the frameworks themselves. In both pyridine and DMF systems, the sample synthesized at 110 °C consistently exhibited the lowest current, indicative of a reduced conductivity (Figure S14). This aligns with prior structural data: at high temperatures, triple bonds degrade, porosity collapses, and conjugation is disrupted. The 40 °C and 75 °C samples, by contrast, showed higher currents and flatter response over time, suggesting that they retain more sp-hybridized carbon and structural continuity necessary for charge transport.

**Figure 4:**
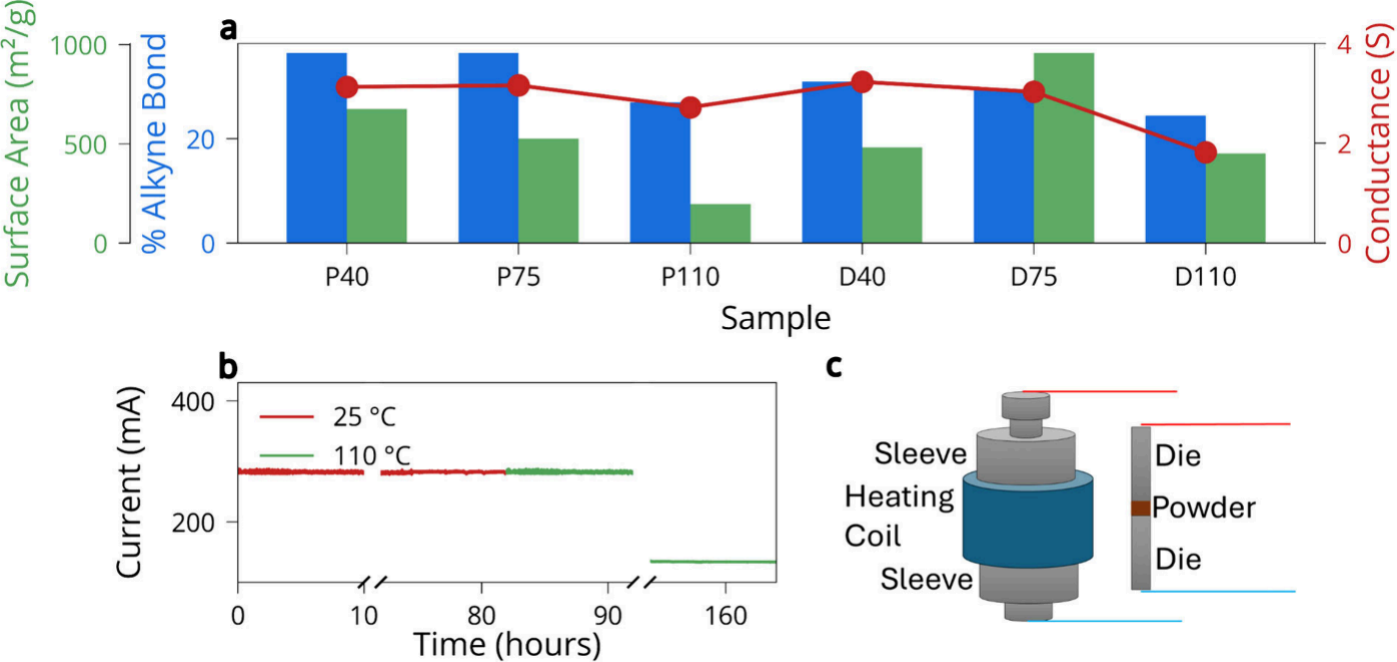
(a) Relative conductance, surface area, and alkyne bond percentage calculated from NMR for each sample. (b) Current stability over time for a sample synthesized in pyridine at 40 °C, held under constant pressure at 25 °C and 110 °C, measured on days 0, 3, and 6. (c) The measurement setup where the red lead is the positive and blue is the negative that is connected to the potentiostat.

To better correlate structure and property, we plotted relative conductance as a function of % alkyne bond and BET surface area, as shown in Figure 4a. The conductance increased with greater alkyne retention, independent of the solvent. This suggests that triple bond preservation is critical for maintaining conductive pathways, likely due to their role in enabling extended π-conjugation and electron delocalization throughout the framework. Notably, once the alkyne content drops to ∼20%, the conductance falls sharply, implying a possible percolation threshold for charge transport. In contrast, no meaningful correlation is observed between surface area and conductance. The sample synthesized in DMF at 75 °C has the highest surface area (958 m^2^/g) but does not exhibit superior conductivity. This supports the conclusion that porosity and accessible surface area do not translate into improved electronic transport, at least under these conditions. Because the samples were measured under the same pressure, the influence of packing density is reduced, and the results more directly reflect the material’s intrinsic ability to conduct charge under compression. These results are further confirmed with the EIS experiments, with the same setup but different electrochemical stimulus (Figure S15). We further assessed the chemical state of any copper catalyst and nitrogen incorporation to determine if impurities cause the conductivity trends. High-resolution Cu 2p XPS spectra show no significant differences in copper content or chemical state across the samples, with all residual copper present in oxidized forms, suggesting a minimal contribution to the observed conductivity trends (Figure S16). In contrast, N 1s spectra reveal increased nitrogen incorporation at higher synthesis temperatures (Figure S17). While nitrogen doping is expected to enhance conductivity, the absence of such an increase indicates that the integrity of the carbon framework is the dominant factor governing electronic transport. The higher conductivity of P110 relative to D110 is consistent with the presence of graphitic nitrogen in P110 (Figure S18).^37^ To confirm that the loss of conductivity observed at high synthesis temperatures is due to irreversible chemical changes and not an artifact of pressure or measurement conditions, we conducted a long-term thermal exposure experiment using a single material. A sample synthesized in pyridine at 40 °C was selected due to its high initial alkyne content and conductivity. The powder was loaded into the pellet press and compressed at 100 MPa as before, and the current was monitored continuously over time while the press was held at a constant temperature.

In the first phase, the press was maintained at 25 °C, and current was recorded for 10 h on day 0 and again after 3 days. As shown in Figure 4b, the current remained stable throughout both time periods, indicating that prolonged pressure alone does not alter conductivity. This suggests that the material’s structure remains chemically and electronically intact under confinement and that there is no pressure-induced degradation or particle rearrangement that would affect transport. In the second phase, the same sample was heated to 110 °C inside the die while still holding the pressure and recording current over 10 h and again 3 days later. The sample initially conducted the same as that at 25 °C, but the conductance significantly dropped by day 6. This behavior suggests that prolonged heating at 110 °C leads to chemical degradation of the alkyne framework, even in the absence of any change in formulation, pressure, or solvent effects. Raman spectroscopy of the recovered sample after the conductivity measurement shows complete disappearance of the alkyne stretching mode at ∼2250 cm^–1^, providing direct evidence of alkyne loss (Figure S19). This structural degradation coincides with the observed decrease in conductivity, confirming that the electrical response is strongly coupled to framework evolution. This result provides direct evidence that alkyne bonds continue to degrade over time at elevated temperatures, leading to irreversible structural changes that disrupt charge transport. It also reinforces the conclusion from the synthesis-temperature series: the key factor limiting conductivity is thermal instability^33^ of the conjugated network.

In conclusion, this study demonstrated that both the reaction temperature and solvent identity play critical roles in determining the chemical, physical, and electronic properties of graphdiyne frameworks. Using hydrogen-substituted graphdiyne as a model system, we show that elevated synthesis temperatures lead to the loss of alkyne bonds, reduced surface area, and diminished conductivity. These changes were captured most clearly by solid-state ^13^C NMR, which reveals structural transformations that are often underrepresented in Raman spectra. Importantly, electrical measurements confirm that conductivity scales with retention of alkyne content and not surface area, underscoring the need to preserve conjugation for electronic function. Together, these findings highlight the importance of careful control of synthetic conditions and rigorous spectroscopic analysis to understand and optimize the structure–property relationships in graphdiyne and related materials.

## Supplementary Material



## Notes

Experimental details, yields, NMR fittings, summary of alkyne ratio, surface area, conductance results, XPS, XRD, setup, and current vs time (PDF)

## Abbreviations

HsGDY: Hydrogen-Substituted Graphdiyne
P40: Pyridine at 40 °C
D40: Dimethylformamide at 40 °C

## References

[1] Li G., Li Y., Liu H., Guo Y., Li Y., Zhu D. (2010). Architecture
of graphdiyne nanoscale films. Chem. Commun..

[2] He F., Li Y. (2023). Advances on Theory
and Experiments of the Energy Applications in
Graphdiyne. CCS Chemistry.

[3] Fu X., Zhao X., Lu T.-B., Yuan M., Wang M. (2023). Graphdiyne-Based
Single-Atom Catalysts with Different Coordination Environments. Angew. Chem., Int. Ed..

[4] Li J., Zhu L., Tung C.-H., Wu L.-Z. (2023). Engineering Graphdiyne for Solar
Photocatalysis. Angew. Chem., Int. Ed..

[5] Zuo Z., Wang D., Zhang J., Lu F., Li Y. (2019). Synthesis
and Applications of Graphdiyne-Based Metal-Free Catalysts. Adv. Mater..

[6] Du H., Zhang Z., He J., Cui Z., Chai J., Ma J., Yang Z., Huang C., Cui G. (2017). A Delicately Designed
Sulfide Graphdiyne Compatible Cathode for High-Performance Lithium/Magnesium–Sulfur
Batteries. Small.

[7] He J., Wang N., Cui Z., Du H., Fu L., Huang C., Yang Z., Shen X., Yi Y., Tu Z. (2017). Hydrogen substituted graphdiyne as carbon-rich
flexible
electrode for lithium and sodium ion batteries. Nat. Commun..

[8] Huang C., Zhang S., Liu H., Li Y., Cui G., Li Y. (2015). Graphdiyne for high capacity and long-life lithium storage. Nano Energy.

[9] Khan K., Tareen A. K., Iqbal M., Shi Z., Zhang H., Guo Z. (2021). Novel emerging graphdiyne based two
dimensional materials: Synthesis,
properties and renewable energy applications. Nano Today.

[10] Li B., Lai C., Zhang M., Zeng G., Liu S., Huang D., Qin L., Liu X., Yi H., Xu F. (2020). Graphdiyne:
A Rising Star of Electrocatalyst Support for Energy Conversion. Adv. Energy Mater..

[11] Yi Y., Huang W., Tian X., Fang B., Wu Z., Zheng S., Li M., Ma H. (2021). Graphdiyne-like Porous
Organic Framework as a Solid-Phase Sulfur Conversion Cathodic Host
for Stable Li–S Batteries. ACS Appl.
Mater. Interfaces.

[12] Greenburg L. C., Gao X., Zhang P., Zheng X., Wang J., Vilá R. A., Cui Y. (2023). Ni Anchored to Hydrogen-Substituted Graphdiyne for Lithium Sulfide
Cathodes in Lithium–Sulfur Batteries. Nano Lett..

[13] Kong S., Cai D., Li G., Xu X., Zhou S., Ding X., Zhang Y., Yang S., Zhou X., Nie H. (2021). Hydrogen-substituted
graphdiyne/graphene as an sp/sp2 hybridized
carbon interlayer for lithium–sulfur batteries. Nanoscale.

[14] Singsen S., Fongkaew I., Hirunsit P., Suthirakun S. (2022). Suppressing
Polysulfides Shuttling and Promoting Sulfur Utilization via Transition
Metal and Nitrogen Co-Doping on Graphdiyne Cathodes of Lithium-Sulfur
Batteries: A First-Principles Modeling. ACS
Applied Energy Materials.

[15] Chen S., Shi G. (2017). Two-Dimensional Materials
for Halide Perovskite-Based Optoelectronic
Devices. Adv. Mater..

[16] Kang J., Huang S., Jiang K., Lu C., Chen Z., Zhu J., Yang C., Ciesielski A., Qiu F., Zhuang X. (2020). 2D Porous
Polymers with sp2-Carbon Connections and Sole sp2-Carbon Skeletons. Adv. Funct. Mater..

[17] Kong Y., Li J., Zeng S., Yin C., Tong L., Zhang J. (2020). Bridging the
Gap between Reality and Ideality of Graphdiyne: The Advances of Synthetic
Methodology. Chem..

[18] Li J., Wang C., Zhang B., Wang Z., Yu W., Chen Y., Liu X., Guo Z., Zhang H. (2020). Artificial
Carbon Graphdiyne: Status and Challenges in Nonlinear Photonic and
Optoelectronic Applications. ACS Appl. Mater.
Interfaces.

[19] Liu J., Chen C., Zhao Y. (2019). Progress and Prospects of Graphdiyne-Based
Materials in Biomedical Applications. Adv. Mater..

[20] Gao X., Zhu Y., Yi D., Zhou J., Zhang S., Yin C., Ding F., Zhang S., Yi X., Wang J. (2018). Ultrathin
graphdiyne film on graphene through solution-phase van der Waals epitaxy. Science Advances.

[21] Gong P., Li J., Wang J., Wu W., Li C., Wang D., Shi J., Liu J., Zhou F., Liu W. (2023). Controlled Growing
of Graphdiyne Film for Friction Reduction and Antiwear. ACS Nano.

[22] He J., Bao K., Cui W., Yu J., Huang C., Shen X., Cui Z., Wang N. (2018). Construction of Large-Area Uniform Graphdiyne
Film for High-Performance Lithium-Ion Batteries. Chemistry A European Journal.

[23] Hu G., He J., Li Y. (2022). Controllable
Synthesis of Two-Dimensional Graphdiyne
Films Catalyzed by a Copper­(II) Trichloro Complex. ACS Catal..

[24] Kong Y., Li X., Wang L., Zhang Z., Feng X., Liu J., Chen C., Tong L., Zhang J. (2022). Rapid Synthesis of
Graphdiyne Films on Hydrogel at the Superspreading Interface for Antibacteria. ACS Nano.

[25] Li J., Li S., Liu Q., Yin C., Tong L., Chen C., Zhang J. (2019). Synthesis of Hydrogen-Substituted
Graphyne Film for Lithium–Sulfur
Battery Applications. Small.

[26] Li J., Xie Z., Xiong Y., Li Z., Huang Q., Zhang S., Zhou J., Liu R., Gao X., Chen C. (2017). Architecture
of β-Graphdiyne-Containing Thin Film Using Modified Glaser–Hay
Coupling Reaction for Enhanced Photocatalytic Property of TiO2. Adv. Mater..

[27] Li X., Huang C., Wang K., Qi L., Zhang C., Zhang M., Xue Y., Cui Y., Li Y. (2023). Alkyne-to-alkene
conversion in graphdiyne driving instant reversible deformation of
whole carbon film. Science Advances.

[28] Li Y., Zhang M., Hu X., Fan X., Yu L., Huang C. (2020). Light and Heat Triggering Modulation
of the Electronic Performance
of a Graphdiyne-Based Thin Film Transistor. J. Phys. Chem. Lett..

[29] Lu C., Yang Y., Wang J., Fu R., Zhao X., Zhao L., Ming Y., Hu Y., Lin H., Tao X. (2018). High-performance graphdiyne-based electrochemical actuators. Nat. Commun..

[30] Zhang C., Xue Y., Zheng X., Qi L., Li Y. (2023). Loaded Cu-Er metal
iso-atoms on graphdiyne for artificial photosynthesis. Mater. Today.

[31] Zhou J., Xie Z., Liu R., Gao X., Li J., Xiong Y., Tong L., Zhang J., Liu Z. (2019). Synthesis
of Ultrathin
Graphdiyne Film Using a Surface Template. ACS
Appl. Mater. Interfaces.

[32] Du R., Zhang N., Xu H., Mao N., Duan W., Wang J., Zhao Q., Liu Z., Zhang J. (2014). CMP Aerogels:
Ultrahigh-Surface-Area Carbon-Based Monolithic Materials with Superb
Sorption Performance. Adv. Mater..

[33] Eeso K., Wygant B. R., Chen Z., Sarswat A., Lambert T. N., Liu N. (2024). The thermal instability of hydrogen-substituted graphdiyne and its
role in lithium–sulfur batteries. Chem.
Commun..

[34] de
Souza F. A. L., Pansini F. N. N., Filho L. F., Ambrozio A. R., Freitas J. C. C., Scopel W. L. (2022). NMR spectral parameters of open-
and closed-shell graphene nanoflakes: Orbital and hyperfine contributions. Carbon.

[35] Wen Y., He K., Zhu Y., Han F., Xu Y., Matsuda I., Ishii Y., Cumings J., Wang C. (2014). Expanded graphite
as
superior anode for sodium-ion batteries. Nat.
Commun..

[36] Hsieh C.-T., Wu W., Eeso K., Chen Z., Leisen J., Filippas A., Lehmann M., Yang G., Liu N. (2025). Unveiling the Reactivity
of Fluoropolymers with Sodium Metal: Mechanistic Insights and Battery
Implications. JACS Au.

[37] Zhuang S., Lee E. S., Lei L., Nunna B. B., Kuang L., Zhang W. (2016). Synthesis of nitrogen-doped graphene catalyst by high-energy wet
ball milling for electrochemical systems. International
Journal of Energy Research.

