# Supplementary material for: Effects of Synthesis Conditions on the Structure and Conductivity of Hydrogen-Substituted Graphdiyne
Source: ACS Mater Au. 2026 Feb 4;6(3):513–8. doi: 10.1021/acsmaterialsau.5c00228 (PMC13177400; doi:10.1021/acsmaterialsau.5c00228)
Supplement: Supplementary file 1 [file mg5c00228_si_001.pdf]

The Effects of Synthesis Conditions on the Structure and Conductivity of Hydrogen Substituted Graphdiyne

Karam Eeso,[a] Akriti Sarswat,[a] Zhitao Chen,[a] Cheng-Tien Hsieh,[a] Timothy N. Lambert,*[b,c] Nian Liu*[a]

[a]School of Chemical and Biomolecular Engineering, Georgia Institute of Technology, Atlanta, GA, 30332, USA

[b]Sandia National Laboratories, Albuquerque, New Mexico, 87185, USA

[c]Center for Integrated Nanotechnologies, Albuquerque, New Mexico, 87185, USA

Experimental Details

Materials

Trimethylsilyl-1,3,5-triethynylbenzene (TMS-TEB), pyridine, DMF, copper(I) chloride (CuCl), chloroform, methanol, and ethanol were purchased from Sigma-Aldrich and used without further purification. All materials were stored under inert conditions when necessary.

Synthesis of Graphdiyne Frameworks

Hydrogen-substituted graphdiyne (HsGDY) was synthesized by Glaser-type oxidative coupling of TMS-protected monomers under varying conditions. In a typical procedure, TMS-TEB (76 mg, 0.2 mmol) and CuCl (6 mg, 0.06 mmol) were dissolved in 2 mL of pyridine/DMF in a glass vial.[35] The vial was sealed and heated at the target temperature (either 40 °C, 75 °C, or 110 °C) for 72 hours under ambient atmosphere. After the reaction, the crude solid was filtered and sequentially washed with pyridine, chloroform, methanol, and ethanol (three times each). The resulting material was soaked in deionized water for 24 hours to facilitate solvent exchange and dried via freeze-drying for another 24 hours. The percent yield is shown in Table S1. Generally the yield does not change within the solvent, but pyridine does have an increased yield compared to DMF, which is most likely due to the decrease in solubility of CuCl.

Characterization

Raman spectroscopy was performed using a Renishaw inVia system with a 785 nm excitation laser. Solid-state ^{13}C MAS-NMR spectra were collected on a Bruker AVIII-HD 300 MHz spectrometer. X-ray photoelectron spectroscopy was performed with a Thermo NEXSA G2 XPS. Peaks were corrected by first fitting the carbon peak and then shifting the right most peak to 284.5 eV. X-ray diffraction (XRD) patterns were recorded using a Rigaku Miniflex II with Cu Kα radiation (λ = 1.5406 Å). Nitrogen physisorption measurements were conducted at 77 K using a Micromeritics Tristar II 3020. Samples were degassed under vacuum (36 Torr) at 40 °C for 12 hours prior to measurement. The BET surface area was calculated using adsorption data in the P/P_0 range of 0.06 to 0.30. TEM was conducted using a FEI Tecnai F30 and SEM was conducted on a Hitachi SU8230.

Bulk electronic conductivity was evaluated using a two-point probe configuration integrated with a hydraulic pellet press. Powder samples were loaded into a 10 mm stainless steel die and compressed to 100 MPa. A fixed voltage of 0.1 V was applied across the press anvils using a BioLogic SP-200 potentiostat, and the resulting steady-state current was recorded over time. Measurements were performed while the sample

remained under pressure to ensure consistent packing and minimize interparticle resistance. For each sample, current was monitored for 20 minutes to ensure stabilization.[36] Relative conductance values were determined by dividing the steady-state current by the applied voltage. Furthermore EIS was applied from 7 MHz to 1 MHz. To assess thermal and temporal stability, a selected sample was held at 25 °C using a PID-controlled cartridge heater attached to the die body, and current was recorded over 10 hours on day 0 and repeated after 3 days. Then same sample was heated to 110°C and the current was again recorded after the temperature was steady and then measured again after 3 days. This setup allowed for direct measurement of pressure-assisted electronic conductance, with minimized contributions from contact resistance, structural rearrangement, or environmental degradation during testing.

Sample	Yield(%)	Carbon (atomic %)
P40	100	94
P75	97	98
P110	98	92
D40	81	95
D75	82	96
D110	78	93

Table S1. Yield of reactions and atomic carbon percentage. The balance for P40 and D40 is silicone while the balance is Nitrogen for the other samples.

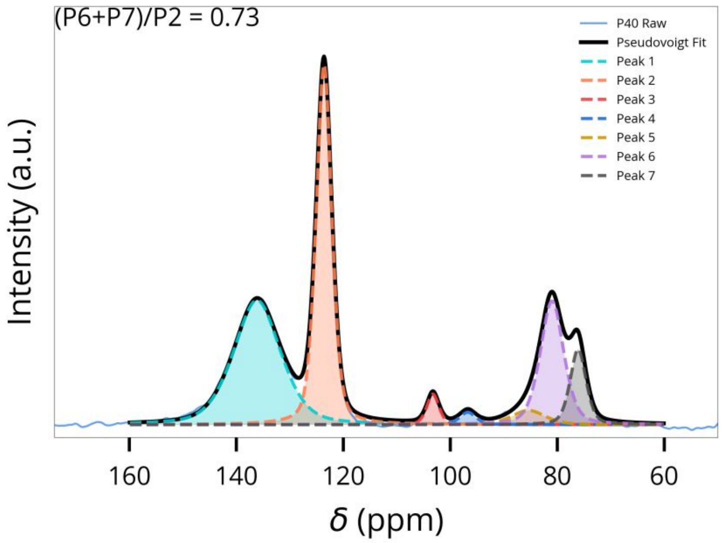

Figure S1. Integration of 40°C Pyridine

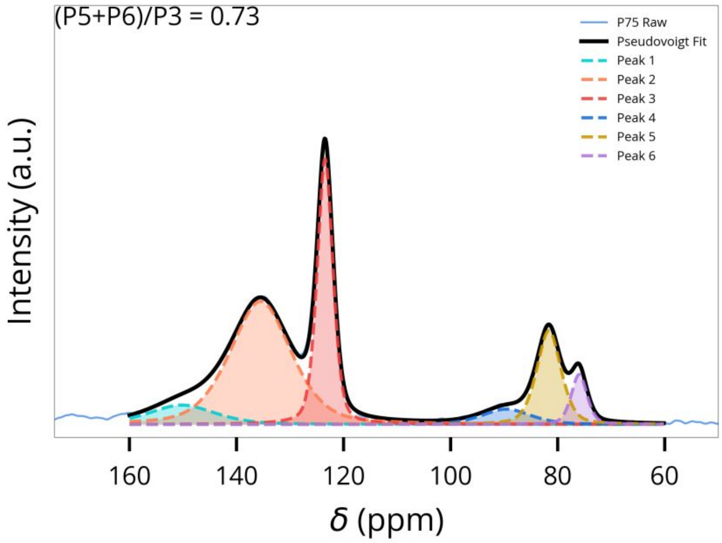

Figure S2. Integration of 75°C Pyridine

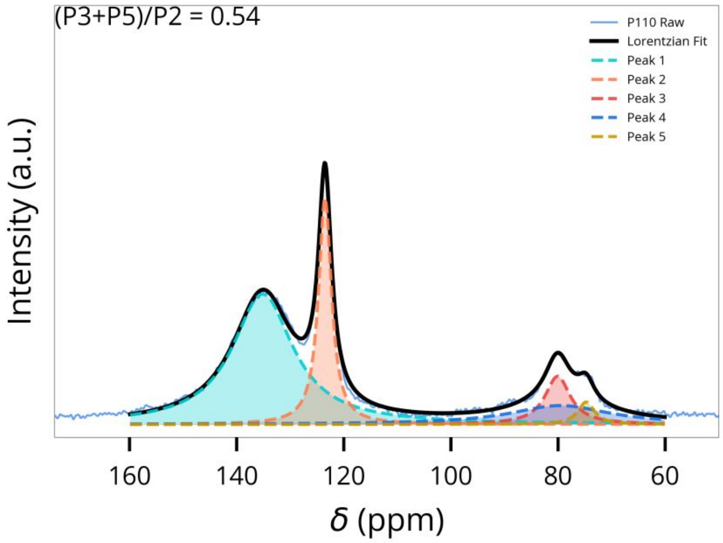

Figure S3. Integration of 110°C Pyridine

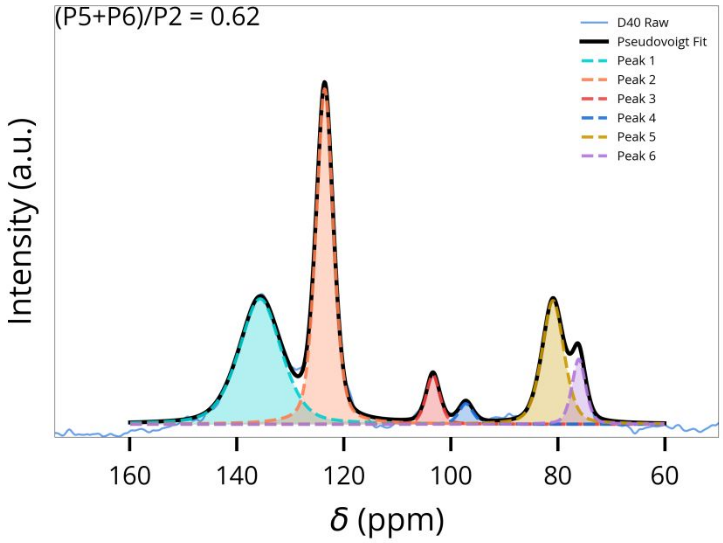

Figure S4. Integration of 40°C DMF

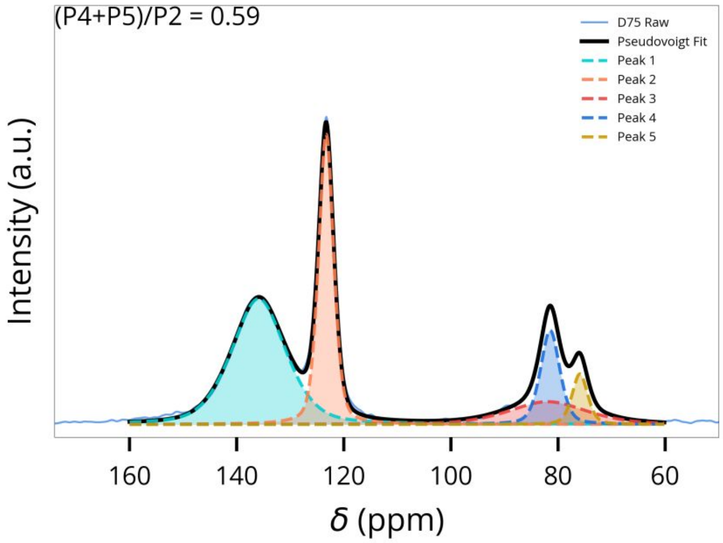

Figure S5. Integration of 75°C DMF

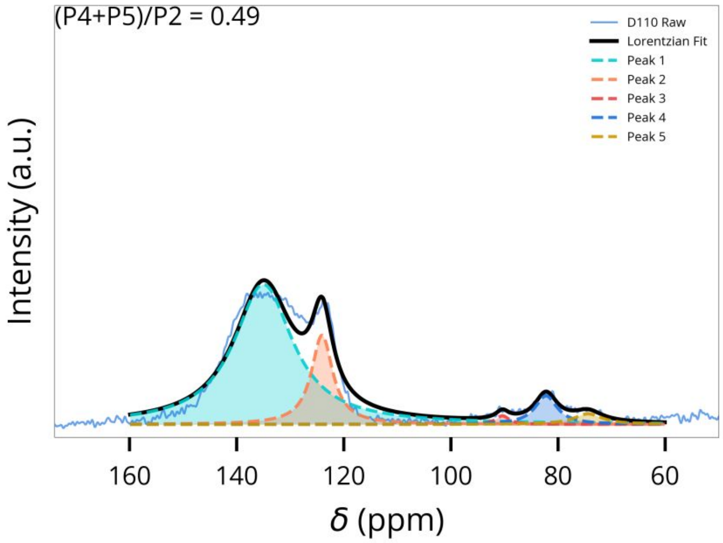

Figure S6. Integration of 110°C DMF

Sample	Alkyne Ratio (NMR)	sp:sp^2 ratio (XPS)	Surface Area (m^2/g)	Conductance (S) (from DC)	Resistance (Ω) (from EIS)
40°C Pyridine	0.37	0.93	676	3.13	.02
75°C Pyridine	0.37	0.54	527	3.16	.028
110°C Pyridine	0.27	0.32	197	2.72	.051
40°C DMF	0.31	0.94	482	3.23	.022
75°C DMF	0.30	0.82	958	3.03	.034
110°C DMF	0.25	0.41	451	1.82	.062

Table S2. Alkyne bond ratio, sp:sp^2 ratio, surface area, conductance and resistance for all samples. The alkyne ratio from the plots is further divided by 2 since we are only dividing by one sp^2 peak and not both

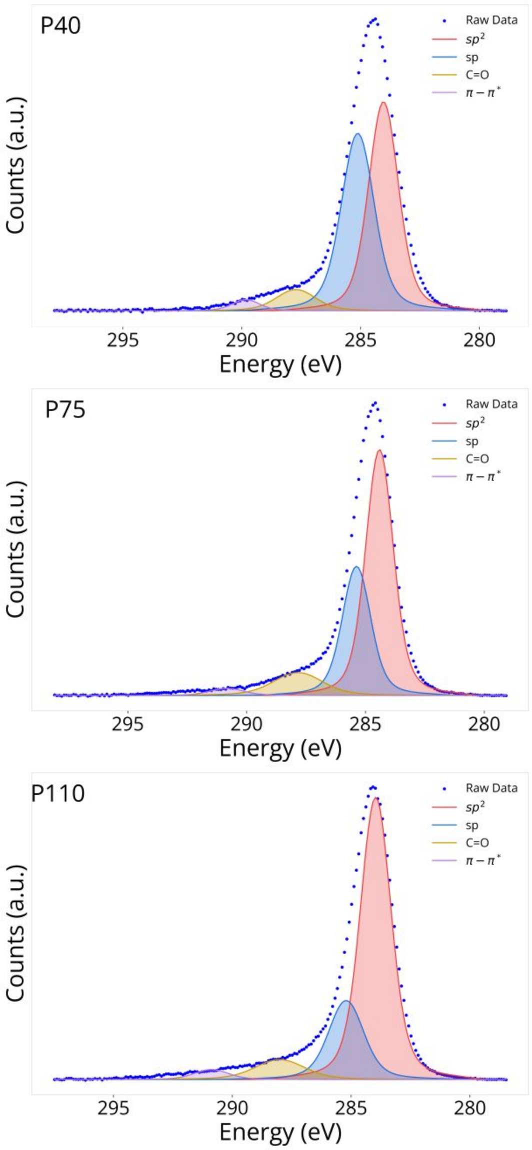

Figure S7. High resolution Carbon XPS results of pyridine samples.

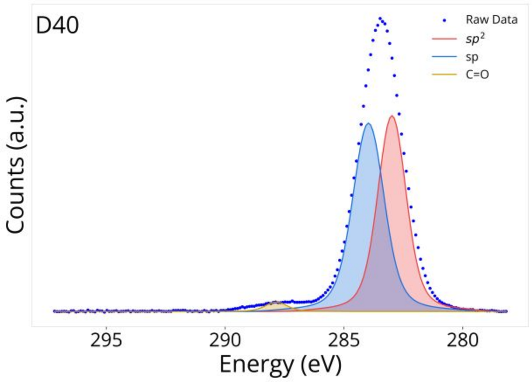

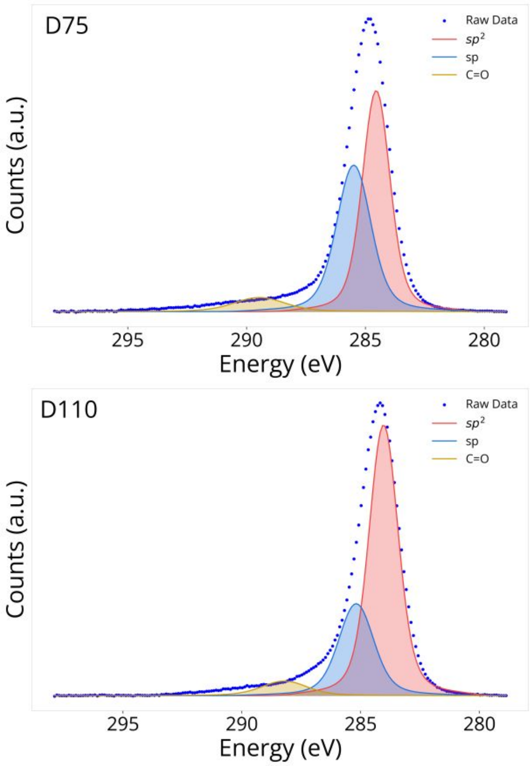

Figure S8. High resolution Carbon XPS results of DMF samples.

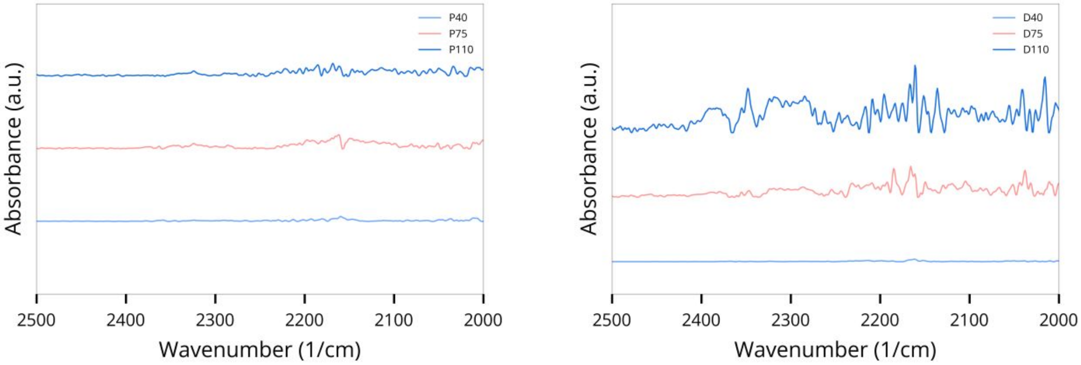

Figure S9. FTIR results for all samples pyridine on the left and DMF on the right

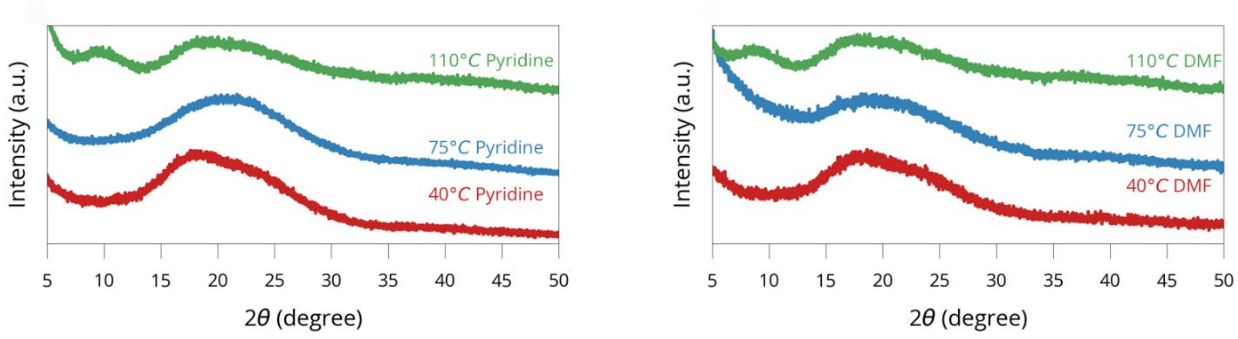

Figure S10. XRD of the Pyridine (left) and DMF (right) samples

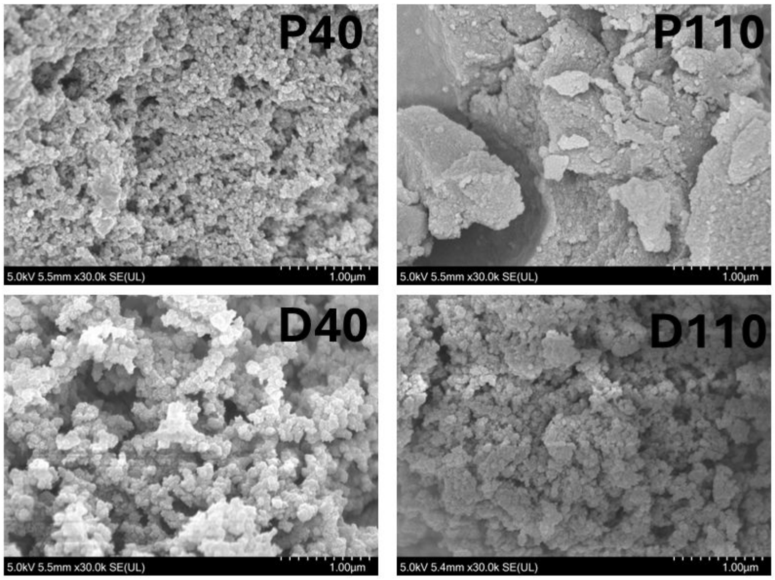

Figure S11. SEM of representative samples

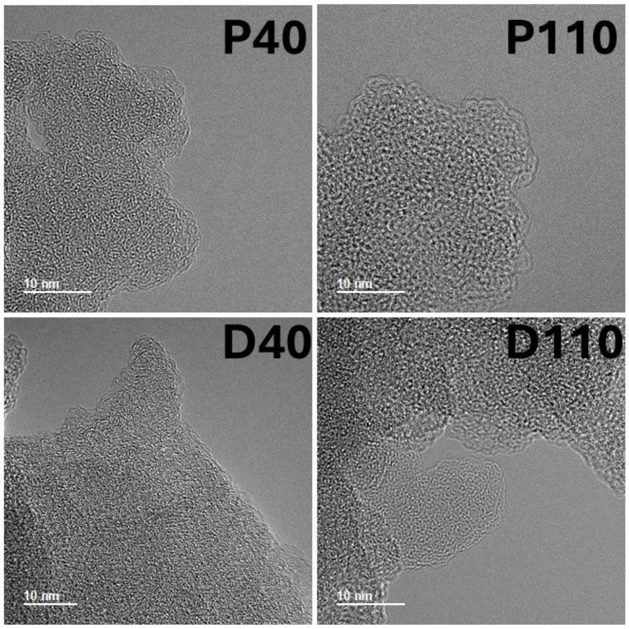

Figure S12. TEM of representative samples

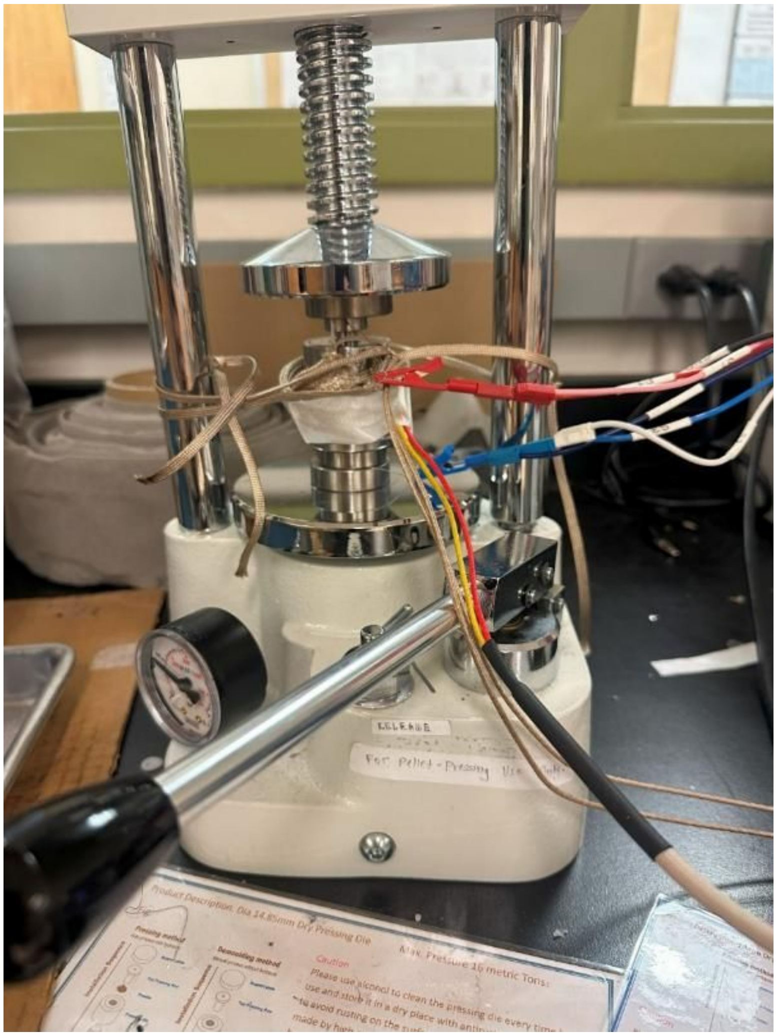

Figure S13. The conductivity measurement setup

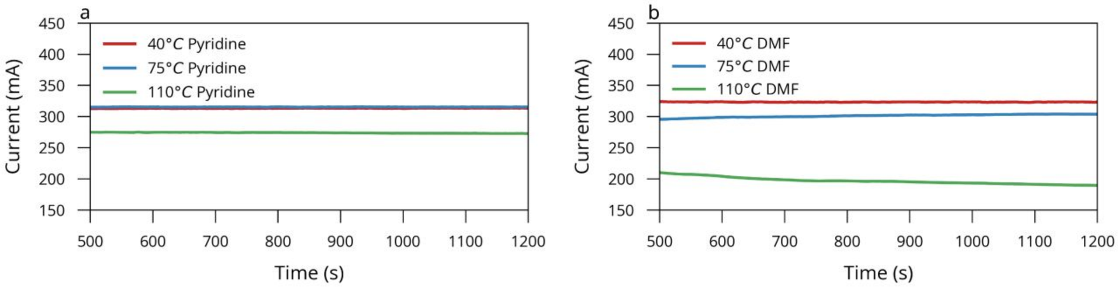

Figure S14. Measured current while applying potential on the different samples synthesized in Pyridine (a) and DMF (b)

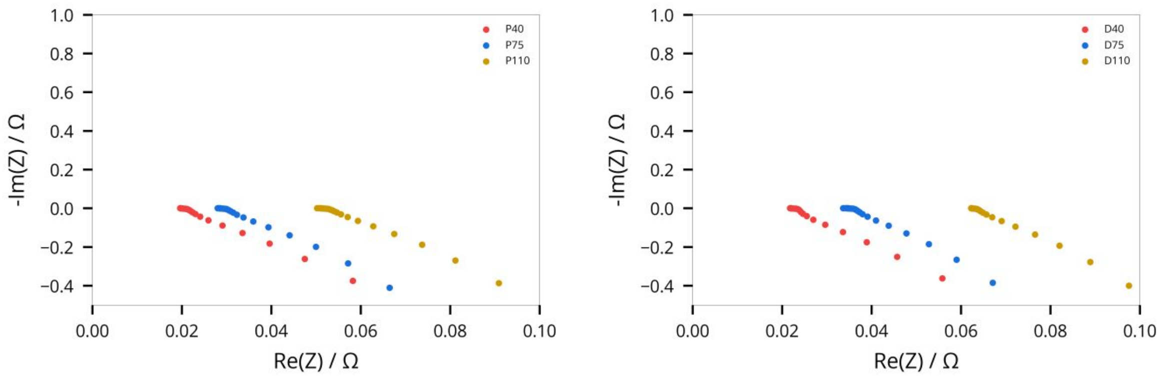

Figure S15. Measured EIS for all samples. It can be seen that the resistance increases with increasing synthesis temperature

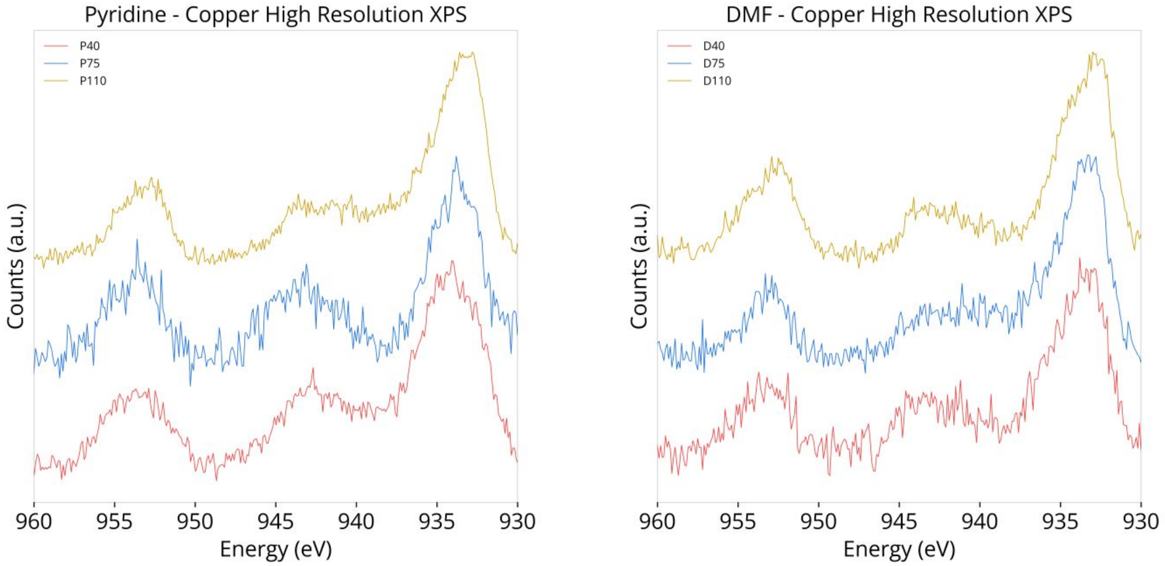

Figure S16. High resolution Cu 2p spectra

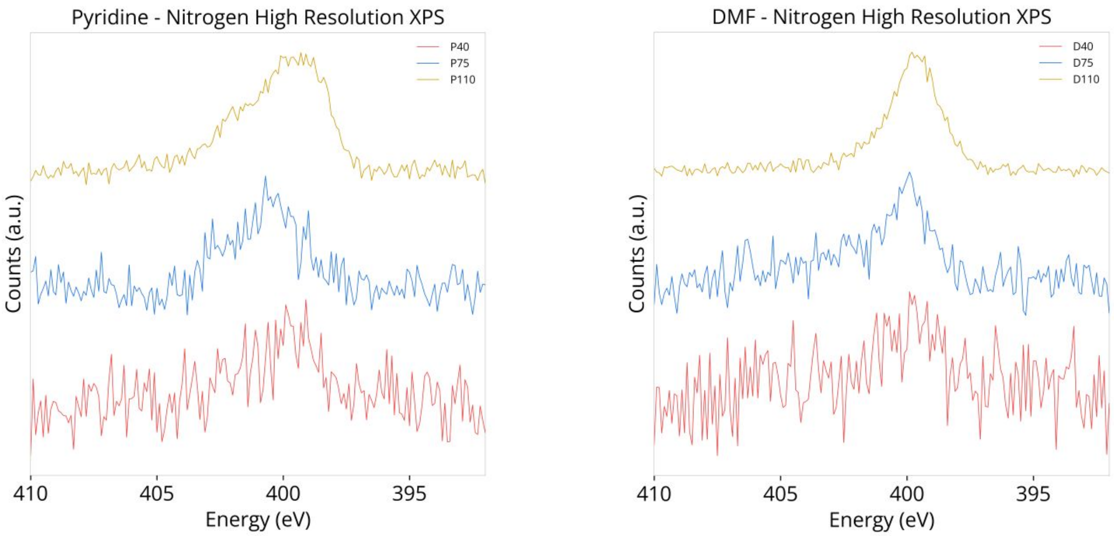

Figure S17 High resolution N 1s spectra

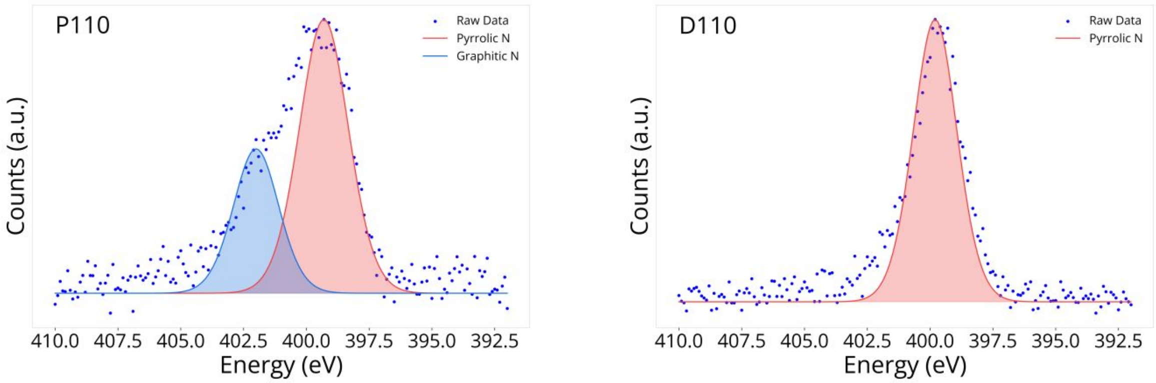

Figure S18. High resolution N 1s spectra deconvoluted for high temperatures

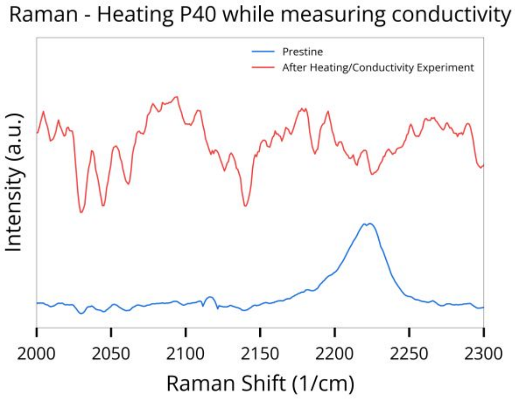

Figure S19. Raman of pristine and after conductivity experiment

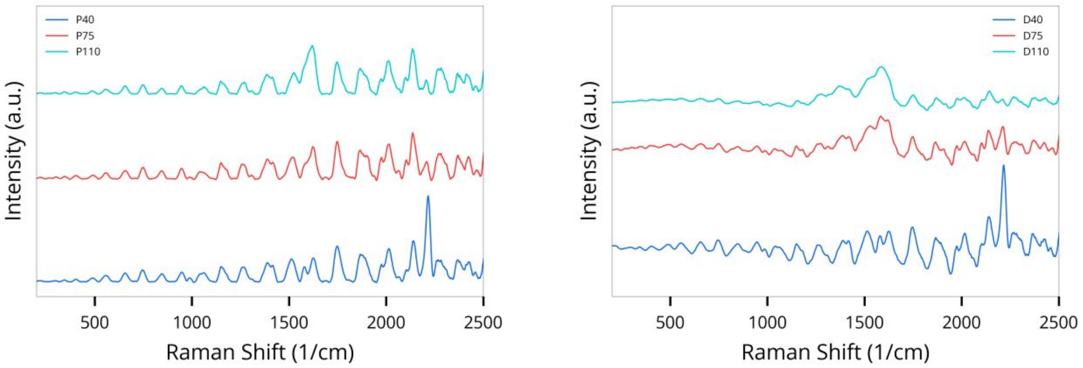

Figure S20. Full Raman spectra showing that the D/G peaks increase with an increase in temperature
